# EEG-cleanse: an automated pipeline for cleaning electroencephalography recordings during full-body movement

**DOI:** 10.1016/j.mex.2025.103702

**Published:** 2025-10-30

**Authors:** Carolina Rico-Olarte, Bjoern M Eskofier, Diego M Lopez

**Affiliations:** a Telematics Department, Universidad del Cauca, Popayán, Colombia; b Machine Learning and Data Analytics Lab, Friedrich-Alexander-Universität Erlangen-Nürnberg, Erlangen, Germany

**Keywords:** Artifact removal, EEG signals, Full-body interaction

## Abstract

Electroencephalography (EEG) data recorded during full-body movement is prone to artifacts that compromise signal quality. We introduce EEG-cleanse, a modular and fully automated preprocessing pipeline for cleaning EEG signals collected in dynamic, real-world contexts. Designed without reliance on specialized hardware, EEG-cleanse was implemented in Python and MATLAB using EEGLAB toolbox. It integrates structured logging and integration of open-source tools:

• Enables automated artifact removal in EEG data recorded during full-body movements.
• Preserves neural signals by combining motion-adaptive preprocessing methods with a hybrid strategy for labeling.
• Demonstrates reproducible performance across immersive exer-learning gaming sessions.

Tested on a dataset of movement-contaminated EEG signals, EEG-cleanse retained over 70 % of recording channels and preserved an average of five brain-related independent components per session. Its performance matches that of a state-of-the-art method without requiring reference sensors, supporting high-quality mobile EEG research in movement-intensive settings. This pipeline enables transparent, reproducible preprocessing for mobile and neuroergonomic EEG studies under naturalistic movement.

## Graphical abstract

**Figure:**
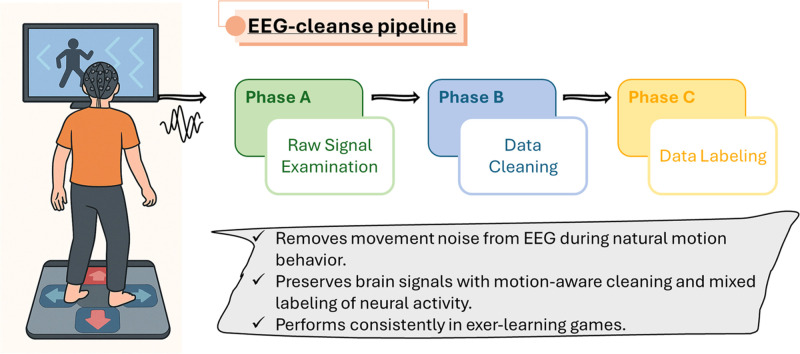

**Specifications Table**

**Table:** 

**Subject area**	Computer Science
**More specific subject area**	EEG preprocessing, artifact removal, neuroergonomics.
**Name of your method**	EEG-cleanse
**Name and reference of original method**	R. J. Downey and D. P. Ferris, “iCanClean Removes Motion, Muscle, Eye, and Line-Noise Artifacts from Phantom EEG,” Sensors, vol. 23, no. 19, Art. no. 19, Jan. 2023, doi: 10.3390/s23198214.
**Resource availability**	Github repository: https://github.com/carolinarico16/EEG-cleanse Python 3.11.9 Python Libraries: json, pandas, numpy MATLAB 2024a EEGLAB 2025.0.0

## Background

EEG signals provide a noninvasive, high-temporal resolution measure of brain electrical activity and serve widely in clinical settings, cognitive contexts, and applied neuroscience. However, these signals are highly vulnerable to contamination from non-neural artifacts, including muscle activity, eye movements, body motion, and environmental interference [1,2]. These artifacts pose significant challenges in studies involving full-body or naturalistic participant movement, where they severely compromise the data quality [1,3].

To mitigate these artifacts, most traditional EEG research constrains participant movement and limit data collection to controlled laboratory settings. However, the field is increasingly shifting toward mobile and ecologically valid paradigms, such as ambulatory monitoring [4], exer-learning gaming [5], and neuroergonomics [6]. These settings demand artifact removal methods that perform reliably under dynamic, motion-rich conditions [7].

Common preprocessing techniques, such as filtering, adaptive filtering using reference sensors, Independent Component Analysis (ICA), and Artifact Subspace Reconstruction (ASR), have functional but limited applicability in motion-rich scenarios. Filtering reduces artifact amplitude but may smear contamination across clean segments [8]. Adaptive filtering assumes consistent, linear projection of noise, which fails in complex motion contexts [9]. ICA requires clean, stationary segments for effective decomposition and is influenced by preprocessing parameters [1,8]. ASR detects statistical outliers but is prone to overcorrection in novel or noisy environments [1,9].

These tools, while valuable, typically lack a better capacity to manage the variability of full-body movement [10]. Many also require manual tuning, external hardware, or task-specific assumptions that reduce generalizability. As a result, the field is missing a preprocessing solution to preserve brain-related components of EEG data recorded in naturalistic contexts [2,3,9].

Recent developments such as dual-layer EEG caps and robotic phantoms enabled artifact benchmarking, but these methods often demand custom equipment and offer limited scalability [11]. Real-time hardware-software frameworks and machine learning approaches (e.g., deep neural networks for artifact detection) show promise. However, these techniques require extensive training data, may lack interpretability, and often depend on system-specific configurations that reduce generalizability [12].

Among recent tools, iCanClean represents a notable advancement [1]. It uses Canonical Correlation Analysis (CCA) to reduce multiple artifact types (motion, ocular, muscular, and line noise). It is also compatible with real-time applications. However, iCanClean functions as a signal-enhancement step rather than a complete pipeline. It lacks synchronization mechanisms, post-processing classification, and task-level integration, which limits its use in complex or event-based EEG signals.

This work checks on the hypothesis that an automated, hardware-independent EEG preprocessing pipeline can preserve neural signal quality in full-body interaction settings as effectively as sensor-dependent methods. Unlike deep learning-based or hybrid artifact removal methods that require large annotated datasets or complex hardware, EEG-cleanse emphasizes interpretability, reproducibility, and hardware independence. EEG-cleanse fills a key methodological gap by providing an open, end-to-end preprocessing framework validated on real human movement data.

## Method details

This section presents the EEG-cleanse pipeline, structured into three sequential phases: (A) Raw Signal Examination, (B) Data Cleaning, and (C) Data Labeling. Each phase is modular, automated, and extensively logged to support transparency and reproducibility. The pipeline integrates tools from Python and MATLAB, using standard libraries and EEGLAB, to transform raw OpenBCI EEG data into clean, brain-labeled datasets.

Phases overview:

1. Phase A: Raw Signal Examination – Preprocessing and segmenting raw EEG data from the OpenBCI software.
2. Phase B: Data Cleaning – Multi-stage cleaning of EEG data using the EEGLAB tool in the MATLAB environment.
3. Phase C: Data Labeling – ICA decomposition and classification of Independent Components (ICs).

**Fig. 1:**
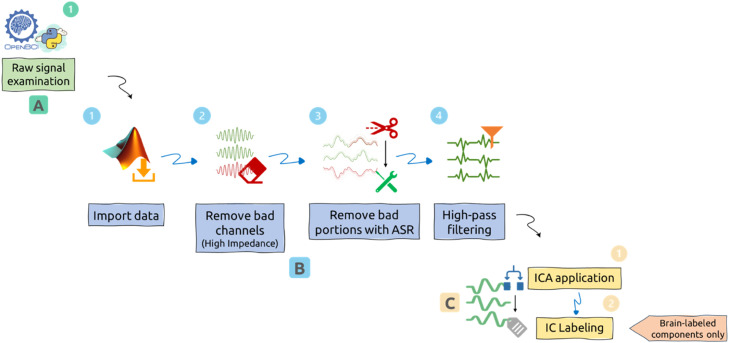
EEG-cleanse pipeline.

Fig. 1 illustrates the full EEG-cleanse pipeline and its modular steps.

### Phase A: raw signal examination

This phase handles the initial preprocessing of raw EEG data collected using OpenBCI hardware. Data was recorded with the *All-in-One EEG Electrode Cap Bundle*,^1^ a device validated for neuroscience research. OpenBCI hardware has been featured in over 400 peer-reviewed publications, lending scientific credibility to its use. The hardware setup included the CytonDaisy board, a 16-channel, Arduino-compatible bioamplifier equipped with a 32-bit processor. The board sampled EEG signals at 125 Hz per channel. The signals were acquired through sintered Ag/AgCl electrodes connected via industry-standard 1.5 mm Touch-Proof connectors.

This phase includes three main modules: format conversion, temporal synchronization with gameplay events, and segmentation into specific cognitive-domain sessions. All procedures run in Python using standard libraries.

### Module A1 – TXT to CSV conversion

The module converts the .txt file, which contains metadata and full EEG data, to a standardized .csv format to simplify handling and ensure compatibility across sessions.

### Module A2 – synchronizing EEG with gameplay

The module aligns EEG signals with gameplay stages recorded in the game’s database. It uses the 'Timestamp' to trim the EEG signal at its start and end, both times taken from the database. It applies a fixed time buffer based on analysis of gameplay timing data to ensure full coverage of each gameplay stage, including preparatory and post-task activity.

### Module A3 – segmenting EEG by mini-games

The module processes each session’s metadata to isolate the EEG signal corresponding to each mini game. It:

• Extracts timestamp information from the .json metadata file.
• Applies custom time adjustments based on the mini game type (according to cognitive domain).
• Saves each segmented EEG as both .csv and .txt files, labeled by cognitive domain and mini game number within the stage.

The phase groups files by experimental condition: GT (baseline with minimal movement) or MVMT (gameplay with full-body interaction) and then by participant.

### Phase B: data cleaning

Phase B of the EEG-cleanse pipeline cleans EEG signals using MATLAB and the EEGLAB toolbox. It imports EEG data from Phase A, identifies and removes low-quality channels, applies automated artifact correction with ASR, and applies high-pass filtering. This phase has four components and ensures that data entering ICA in Phase C is as clean and standardized as possible.

### Component 1: import data

The component transforms EEG recordings from Phase A into EEGLAB-compatible datasets through two modules.

#### Module B1.1 – data preparation

The module:

1. Transposes the data to match MATLAB's column-major data format.
2. Saves the transposed data into .mat files, labeled with a participant-specific identifier.

#### Module B1.2 – conversion to eeglab format

The module imports the .mat files into EEGLAB and converts them into .set format. During this step, the pipeline assigns channel metadata using the predefined channel location file of OpenBCI software and validates the integrity of the channel configuration.

### Component 2: remove bad channels

EEG-cleanse identifies and removes noisy or unreliable channels through two modules.

#### Module B2.1 – high-impedance channel removal

Channels with extreme voltage values are considered to have poor electrode contact and are removed. EEG-cleanse evaluates each channel for high impedances and flags them. The threshold (>48,000 µV mean) is determined from the distribution of channel amplitudes across signals.

#### Module B2.2 – flatline, low-correlation, and noisy channels

The module applies EEGLAB metrics to identify and remove flatline channels (no signal variance for ≥5 s), low-correlation channels (correlation with the robust signal mean below 0.85), and noisy channels (z-score-based amplitude outliers exceeding a threshold of 4).

### Component 3: remove bad portions with ASR

The third component of the data cleaning phase applies ASR to detect and correct transient, high-amplitude artifacts in the EEG signal. ASR is a data-driven algorithm that identifies abnormal segments by comparing them to a statistical model derived from clean EEG data [13]. Unlike fixed-template approaches, ASR adapts to the noise characteristics of each session, making it suited for recordings with high variability, such as those involving full-body movement.

To configure ASR, the pipeline uses the following parameters:

• ChannelCriterion = 0.85 removes channels with low correlation to the robust signal average
• LineNoiseCriterion = 4 removes channels with excessive line noise
• BurstCriterion = 3 sets the aggressiveness of artifact detection
• WindowCriterion = 0.05 flags time windows with pervasive noise

The quality of the clean reference used for ASR calibration varies across sessions. This step is critical for minimizing movement-related artifacts that traditional filtering does not address. By calibrating ASR individually for each session and applying targeted correction, EEG-cleanse preserves the integrity of neural signals while eliminating transient contamination in a reproducible and fully automated manner.

### Component 4: high-pass filtering

The final component of the data cleaning phase applies a high-pass finite impulse response (FIR) filter [14] to all EEG signals. This step removes low-frequency noise, including baseline drift and DC offsets, which can distort the signal and interfere with subsequent ICA decomposition. The pipeline adjusts the cutoff frequency dynamically to accommodate differences in recording length. Shorter sessions use a 0.5 Hz cutoff, while longer sessions use a 1.0 Hz cutoff, optimizing the trade-off between low-frequency artifact removal and preservation of slow neural dynamics. All filtering uses zero-phase FIR filtering with a Hamming window, with a fixed filter order of 208 to balance spectral precision and computational efficiency.

The pipeline positions high-pass filtering after ASR, based on theoretical reasoning and empirical validation. Although ASR requires zero-mean, drift-free input [15], applying a high-pass filter too early can smear brief, high-amplitude artifacts across time [16]. This risk increases with sharp transition-band filters, short datasets, or aggressive ASR thresholds. We tested low-cutoff filtering (0.25–0.5 Hz) before ASR. As the cutoff decreased, correlation with the original signal improved, indicating minimal signal distortion. However, to avoid even subtle artifact spreading, the pipeline runs ASR directly on channel-cleaned, unfiltered data. High-pass filtering is then applied afterward to remove remaining low-frequency drift and prepare the cleaned signal for ICA decomposition. This ordering ensures ASR operates on the most authentic signal possible while still meeting denoising goals.

### Phase C: data labeling

Phase C of the EEG-cleanse pipeline performs ICA and automated labeling of ICs to isolate brain-related signal sources. The objectives of this phase are to apply ICA process and use ICLabel classification to discard non-brain signals. All processing runs in MATLAB using the EEGLAB toolbox and its associated plugins, including ICLabel [17]. This phase completes the preprocessing workflow by producing a curated set of neural components.

### Component 1: ICA application

This component has two modules.

#### Module C1.1 – compute ICA

The pipeline performs ICA on all EEG signals cleaned and filtered during Phase B. For each session, it computes ICA separately on two versions of the dataset:

• The ASR-cleaned dataset
• The high-pass filtered dataset

This dual-decomposition approach provides flexibility in downstream processing allowing ICA results to be reused across multiple data representations. It also supports a robust component labeling strategy in the next component.

Before decomposition, the pipeline centers each signal and reshapes it into a two-dimensional matrix (channels × time). It then runs ICA using EEGLAB’s runica function in extended mode. The pipeline saves the resulting weight and sphere matrices, along with the decomposed signals, in a structured output folder.

#### Module C1.2 – apply ICA

This module applies ICA matrices from the previous module to different versions of the original EEG signals. By transferring ICA decompositions across conditions, the pipeline enables comparative analysis to improve the IC classification reliability.

For each session, it performs the following ICA-to-signal mappings:

• Clean-to-clean
• Filtered-to-filtered
• Filtered-to-clean

After loading a target signal, the pipeline assigns the corresponding ICA matrices to the EEG structure, recalculates the inverse unmixing matrix, and embeds all ICA-related fields (weights, sphere, activations) into the signal. It then saves each resulting file with a name specifying source–target combination.

### Component 2: IC labeling

This component classifies ICs automatically using the ICLabel toolbox [17]. ICLabel assigns each component a probability of belonging to predefined categories: Brain, Muscle, Eye, Heart, Line Noise, Channel Noise, and Other. It is selected for its validated performance and integration with EEGLAB, outperforming other available IC labeling tools in reliability and usability.

The pipeline evaluates each IC across up to three ICA-applied versions of the same EEG signal and applies a hybrid voting-based decision strategy to decide whether to retain it as brain-related. The strategy retains a component as “brain” if it meets any of the following criteria:

1. Classified as Brain in at least two versions with probability ≥ 0.6.
2. One version shows high-confidence Brain classification (probability ≥ 0.7) and others do not contradict it.
3. The weighted average Brain probability across all versions exceeds a conservative threshold.
4. Most versions agree on the Brain label with moderate confidence.

If a component fails to meet any criterion, the pipeline marks it for removal. It labels all retained components as brain-related and saves the final signals containing only these ICs.

## Method validation

We designed and tested EEG-cleanse using 308 EEG signals from 14 participants collected with OpenBCI hardware during full-body interaction with the HapHop-Physio system [11]. Each session comprised a still baseline (GT) and an active movement stage (MVMT). Two recordings were excluded due to data integrity issues. The remaining 306 included 82 GT and 224 MVMT sessions.

We tested EEG-cleanse through internal validation across key stages of the pipeline to determine its effectiveness in preserving a usable EEG signal while removing artifacts caused by full-body movement. We computed metrics for three areas: (1) channel-level signal after cleaning in Phase B, (2) artifact correction efficiency when applying ASR, and (3) preservation of brain-related ICs after ICA decomposition and classification.

The evaluation used internal metrics linked to signal quality and artifact suppression including spectral entropy, frequency band power, correlation to raw data, and IC classification rates. These metrics were chosen for their relevance to signal complexity, cognitive interpretability (for HapHop-Physio purposes), and benchmarking preprocessing fidelity in the absence of task-based EEG labels.

1. Channel Cleaning Efficiency

Across 306 sessions (16 electrodes by EEG signal), we recorded 4896 channels. After applying the two-step cleaning process, which first removed high-impedance channels followed by flatline, low-correlation, and noisy channels, 3520 channels were retained, corresponding to a channel retention rate of 71.43 % (Table 1). One session was excluded due to the insufficient number of clean channels.

**Table 1:** Channel retention after high-impedance and noise-based filtering.

Step	Channels Retained	% of Original
After High-Impedance Filtering	4075	82.67 %
After Flatline/Noise Filtering	3520	71.43 %

High-impedance and noisy channel removal ensures that only reliable electrodes contribute to ICA decomposition, which is critical for accurate artifact separation in motion-rich recordings.

2. ASR Effectiveness

We applied ASR to the remaining 305 sessions. On average, 83.36 % of each session was identified as suitable for the clean reference signal used by ASR. In 102 sessions, ASR identified fully clean data and performed no reconstruction. Across all sessions, only 20.97 % of the data was reconstructed on average, indicating that ASR acted selectively and did not overcorrect the signal.

These results show that EEG-cleanse preserves large portions of clean neural data during ASR while selectively suppressing contaminated segments. The low reconstruction rate confirms that ASR avoids unnecessary signal alteration.

3. ICA Decomposition and Brain Component Retention

We performed ICA on 290 sessions; 15 sessions were excluded due to insufficient signal variance or short recording duration after cleaning. ICA decomposition produced 3461 ICs across sessions, of which, 2440 components (70.5 %) were retained as brain-related. Five sessions (1.7 %) failed to yield any usable brain-related ICs and were excluded from the final version of the signal (Table 2).

**Table 2:** ICA decomposition results and brain-related component retention.

Metric	Value
Sessions after filtering	305
Sessions included in ICA	290
Sessions retained after IC labeling	285
Total ICs generated	3461
Brain ICs retained	2440
Brain IC retention rate	70.5 %

On average, each session retained approximately five brain-classified ICs, showing effective preservation of plausible neural sources in motion-rich environments. These findings demonstrate that the pipeline isolates neurophysiologically meaningful signal components despite substantial motion-related contamination.

### Performance of the canonical approach against the new approach

We benchmarked EEG-cleanse against iCanClean, a CCA-based EEG cleaning strategy. While iCanClean was originally validated using a phantom-head model mounted on a robotic platform capable of simulating walking-related and multi-source artifacts, its evaluation was limited to controlled setups with predefined contamination profiles. In contrast, we developed EEG-cleanse for and tested it on EEG data recorded during real human movement in naturalistic, full-body interactive tasks.

#### Evaluation setup

We applied both pipelines (EEG-cleanse and iCanClean) to the same dataset of 308 EEG signals collected like described before. To ensure a fair comparison, we processed raw EEG data independently through each pipeline and applied all downstream steps (including ICA and IC classification using ICLabel) consistently across both methods.

Because iCanClean does not include built-in ICA or component classification, we manually applied the ICA and ICLabel under similar conditions used to EEG-cleanse (see Phase C). Specifically:

• We computed ICA using EEGLAB’s runica algorithm in extended mode
• We used ICLabel for automatic component classification.
• We retained only ICs with a “brain” label probability ≥ 0.7.
• We reconstructed EEG signals from the retained brain-only components.

To enable meaningful comparisons despite differences in signal representation (iCanClean outputs data in component space EEG-cleanse in channel space), we applied two types of post-cleaning normalization:

• Z-score normalization (mean = 0, std = 1) to assess relative power, spectral entropy, and signal similarity to the original signal
• L2 normalization to evaluate energy structure and waveform coherence

#### Evaluation metrics

We computed two classes of evaluation metrics at two points in the pipeline: (1) after the cleaning stage (Phase B of EEG-cleanse vs. iCanClean output), and (2) after IC classification and signal reconstruction from brain-labeled components.

### Post-cleaning (unit-invariant) metrics

• Spectral Entropy: uniformity of the power spectrum.
• Alpha/Beta Power: relative power in canonical EEG frequency bands.
• SNR Proxy: ratio of alpha power to total power, serving as a proxy for signal quality.
• Correlation to Raw: Pearson correlation with unprocessed EEG.
• Matrix Rank: effective dimensionality of the cleaned signal

### ICLabel-based metrics

• Brain Fraction: mean “brain” probability across retained ICs.
• Artifact Fraction: total non-brain probability.
• Number of Brain ICs: ICs retained with brain probability ≥ 0.7.
• Artifact IC Count: retained ICs classified as non-brain.

#### Results of the comparison

##### Z-score normalization

We computed all metrics on z-scored data (except rank, which is scale-invariant). Results are in Table 3.

• **Samples:** 580 comparisons (290 sessions × 2 methods)
• **Normalization:** channel-wise z-scoring across time

**Table 3:** Metrics results from z-score normalization.

Metric	iCanClean Mean	EEG-cleanse Mean	t-statistic	p-value	Cohen's d	Significant?
Spectral Entropy	8.619	8.577	0.63	0.532	0.03	No
Alpha Power	0.079	0.076	0.77	0.442	0.03	No
Beta Power	0.138	0.102	6.94	2.6e-11	0.29	Yes
SNR Proxy	0.264	0.200	11.36	5.8e-25	0.47	Yes
CorrelationToRaw	0.202	0.153	4.38	1.65e-5	0.18	Yes
Rank	12.086	12.086	NaN	NaN	NaN	(No diff)

##### L2 normalization

We computed Welch-based band power and correlation using raw EEG data normalized by L2 norm Table 4.

• **Samples:** 580 (same structure)
• **Normalization:** channel-wise L2 normalization across time

**Table 4:** Metrics results from L2 normalization.

Metric	iCanClean Mean	EEG-cleanse Mean	t-statistic	p-value	Cohen's d	Significant?
Retained Variance	2265.50	2265.88	−3.65	3.07e-4	0.21	Yes
Spectral Entropy	8.620	8.577	0.64	0.524	0.04	No
Alpha Power	9e-6	8e-6	2.63	0.0089	0.15	Yes
Beta Power	1.6e-5	1.2e-5	9.94	3.23e-20	0.57	Yes
SNR Proxy	0.094	0.067	12.36	1.89e-28	0.71	Yes
CorrelationToRaw	0.202	0.153	4.38	1.65e-5	0.25	Yes
Rank	12.086	12.086	NaN	NaN	NaN	(No diff)

#### Summary of results

• Brain Fraction was higher in iCanClean (∼91.2 %), but this coincided with zero retained artifact ICs, which is atypical for real-world EEG and may suggest over-suppression of borderline components.
• EEG-cleanse retained a more heterogeneous IC profile, including a small number of artifact components, reflecting a realistic EEG composition.
• The number of retained brain ICs was equivalent across methods (mean ≈ 5, *p* > 0.2).
• iCanClean outperformed EEG-cleanse on SNR proxy and correlation to raw signals, with medium effect sizes (Cohen’s d ≈ 0.47–0.51), suggesting greater waveform preservation and reduced apparent noise.
• No significant differences were found in spectral entropy, alpha power or matrix rank (d < 0.2), indicating that both pipelines preserved signal complexity and dimensionality similarly.

These findings suggest that iCanClean offers strong artifact suppression and waveform fidelity in controlled or simulated contexts. EEG-cleanse achieves comparable channel retention and brain IC preservation while retaining a small fraction of artifact ICs, representing a trade-off between artifact removal and signal preservation Table 5.

**Table 5:** EEG-cleanse vs iCanClean.

Feature	EEG-cleanse	iCanClean
Automation	Fully automated	Semi-automated
Hardware required	No extra sensors	Dual-layer optional
Component classification	Hybrid/voting ICLabel	Manual/none
Real-world tested	Yes (exer-learning game)	No (phantom-based)
Channels retained	∼71 %	-
Brain ICs retained	∼5/session	∼5/session

While iCanClean suppresses more non-brain components, its zero-retained artifact ICs suggest aggressive filtering that may remove ambiguous but valid signals. EEG-cleanse preserves a small number of mixed ICs, offering realistic EEG composition under naturalistic movement. This difference reflects a trade-off: iCanClean prioritizes purity, whereas EEG-cleanse emphasizes balanced preservation for applications needing full signal context.

### Advantages of the EEG-cleanse method and practical tips

The comparative analysis outlined above highlights key advantages of EEG-cleanse over conventional preprocessing methods, particularly when applied to EEG data recorded during full-body, real-world interaction. Although EEG-cleanse was validated on 16-channel OpenBCI data, the modular structure supports straightforward adaptation to high-density EEG caps (e.g., 64 or 128 channels). Channel configuration files and preprocessing parameters (e.g., impedance thresholds, ASR calibration) can be adjusted via the configuration script. Also, EEG-cleanse maintains temporal precision and spectral structure suitable for event-related potential (ERP) and time–frequency analyses. Its preservation of mixed ICs supports downstream cognitive or neuroergonomic tasks where motion and neural activity co-occur. EEG-cleanse was developed to meet transparency, flexibility, and ecological validity as core design goals.

Full Transparency and Modularity: each step is explicitly defined and tunable.

Real-World Validation: tested in real human interaction.

Balanced Signal Preservation: avoids aggressive removal of borderline ICs.

Hardware-Independent and Flexible: requires no auxiliary sensors

Extensible Design: compatible with additional tools (e.g., CCA modules)

## Limitations

• EEG-cleanse is designed for signals with moderate to high temporal alignment between EEG and behavioral data; poor synchronization may reduce ICA labeling accuracy.
• It requires a minimum session length and data quality threshold to perform ICA decomposition; very short or noisy recordings are excluded.
• Although ASR and ICLabel are automated, minor tuning of parameters is sometimes necessary for data recorded under substantially different hardware or sampling rates.
• It is not evaluated for clinical EEG or infant recordings; performance in those domains remains unverified.
• EEG-cleanse is validated only on OpenBCI 16-channel signals. Its modular design supports adaptation to higher-density montages or other EEG systems, but generalizability is untested. Portability to other hardware and sampling rates will be explored in future studies.
• Current validation uses only signal-level metrics. Task-relevant benchmarks such asERP, classification accuracy in cognitive tasks, and inter-rater IC labeling comparisons are not yet included.

## Ethics statements

All participants provided written informed consent prior to participation in accordance with the Declaration of Helsinki. The study protocol, including data collection procedures involving EEG and full-body interaction, was approved by the institutional ethics review board of Universidad del Cauca, according to the minute document 6.1 - 1.25/53 from June 30th, 2020.

## CRediT authorship contribution statement

**Carolina Rico-Olarte:** Conceptualization, Methodology, Software, Validation, Writing – original draft. **Bjoern M Eskofier:** Writing – review & editing, Supervision. **Diego M Lopez:** Methodology, Writing – review & editing, Supervision.

## Declaration of competing interest

The authors declare that they have no known competing financial interests or personal relationships that could have appeared to influence the work reported in this paper.

## Data Availability

Data will be made available on request.

## Footnotes

1 https://shop.openbci.com/products/all-in-one-eeg-electrode-cap-bundle
